# Early progression of Krabbe disease in patients with symptom onset between 0 and 5 months

**DOI:** 10.1186/s13023-019-1018-4

**Published:** 2019-02-18

**Authors:** Maria L. Beltran-Quintero, Nicholas A. Bascou, Michele D. Poe, David A. Wenger, Carlos A. Saavedra-Matiz, Matthew J. Nichols, Maria L. Escolar

**Affiliations:** 1 Program for the Study of Neurodevelopment in Rare Disorders and Center for Rare Disease Therapy, UPMC Children’s Hospital of Pittsburgh, University of Pittsburgh, 4401 Penn Avenue, Pittsburgh, PA 15144 USA; 2 Sidney Kimmel Medical College, 1020 Locust St, Room 346, Philadelphia, PA 19107 USA; 3 Division of Genetics, Newborn Screening program, Wadsworth Center, New York State Department of Health, Albany, NY USA

**Keywords:** Krabbe disease, Globoid cell leukodystrophy, Early-infantile, Infantile, Natural history, Newborn screening

## Abstract

**Background:**

Krabbe disease is a rare neurological disorder caused by a deficiency in the lysosomal enzyme, β-galactocerebrosidase, resulting in demyelination of the central and peripheral nervous systems. If left without treatment, Krabbe disease results in progressive neurodegeneration with reduced quality of life and early death. The purpose of this prospective study was to describe the natural progression of early onset Krabbe disease in a large cohort of patients.

**Methods:**

Patients with early onset Krabbe disease were prospectively evaluated between 1999 and 2018. Data sources included diagnostic testing, parent questionnaires, standardized multidisciplinary neurodevelopmental assessments, and neuroradiological and neurophysiological tests.

**Results:**

We evaluated 88 children with onset between 0 and 5 months. Median age of symptom onset was 4 months; median time to diagnosis after onset was 3 months. The most common initial symptoms were irritability, feeding difficulties, appendicular spasticity, and developmental delay. Other prevalent symptoms included axial hypotonia, abnormal deep tendon reflexes, constipation, abnormal pupillary response, scoliosis, loss of head control, and dysautonomia. Results of nerve conduction studies showed that 100% of patients developed peripheral neuropathy by 6 months of age. Median galactocerebrosidase enzyme activity was 0.05 nmol/h/mg protein. The median survival was 2 years.

**Conclusions:**

This is the largest prospective natural history study of Krabbe disease. It provides a comprehensive description of the disease during the first 2 years of life. With recent inclusion of state mandated newborn screening programs and promising therapeutic interventions, enhancing our understanding of disease progression in early onset Krabbe disease will be critical for developing treatments, designing clinical trials, and evaluating outcomes.

## Background

Krabbe disease, or globoid cell leukodystrophy (GLD; OMIM **#** 245200), is a rare neurological disease with an autosomal recessive inheritance pattern. Mutations in the *GALC* gene, located on chromosome 14, cause a deficiency of the lysosomal enzyme β-galactocerebrosidase [1]. The resulting accumulation of the intermediates, galactosylceramide and galactosylsphingosine (psychosine), leads to severe demyelination of both the central (CNS) and peripheral (PNS) nervous systems [2–4]. Patients with early onset typically have a more severe and rapidly progressing phenotype than patients with later onset. Common symptoms of early onset Krabbe disease include appendicular spasticity, axial hypotonia, irritability, severe psychomotor delay or regression, seizures, and premature death [5–8], whereas clinical features of later onset Krabbe disease generally include gradual psychomotor regression, development of progressive spastic paraparesis, and gait abnormalities [9–11].

The incidence of Krabbe disease has been estimated as one in 100,000 births in the United States and Europe [1, 12]. Historically, patients with Krabbe disease have been divided into subtypes based on their age of onset: early-infantile (0–5 months), late-infantile (6–36 months), juvenile (3–16 years), and adult (> 16 years) [13–15]. However, despite these long-established conventions in nomenclature, few standardized natural history studies of Krabbe disease have been conducted to define the phenotypes and the viability of these arbitrary subtypes remains to be tested by a rigorous, prospectively designed protocol. Currently, the only treatment available for Krabbe disease is hemopoietic stem cell transplantation (HSCT). HSCT works by introducing donor derived cells that migrate to the CNS, providing compensatory function for defective GALC. HSCT has been most successful in asymptomatic children by stabilizing or slowing the progression of CNS demyelination. Outcomes in patients who are already symptomatic have been comparatively worse [16–20].

Newborn screening (NBS) for Krabbe disease was introduced in New York State in 2006 and then it has been mandated in 9 additional states out of which 6 are currently screening (NY, OH, MO, KY, IL, TN). The correlations between GALC activity, psychosine levels, genetic variations, and clinical presentation are not well established, which can cause difficulty for parents and physicians when they are making decisions regarding treatment with HSCT [21–25]. As a result, newborns determined to be at risk of developing disease must be extensively monitored throughout their early years of life to identify early clinical markers of disease. Therefore, knowledge of the natural progression of early onset Krabbe disease is needed in order to help families and clinicians make informed treatment decisions as NBS continues to expand throughout the United States. In addition, natural history data will be instrumental in evaluating the efficacy of new treatments, such as gene therapy and enzyme replacement therapy [26].

While studies describing the clinical presentation of Krabbe disease have been published to date, most are based on registry data or retrospective analysis of patient records and are therefore subject to numerous limitations including incomplete datasets, inconsistent non-standardized observations, and small sample sizes. The first case series describing the clinical presentation was published by Knud Krabbe in 1916 [27]. The article described patients with infantile onset as having irritability, feeding difficulties and as the disease progressed, hypertonicity, seizures, loss of vision and hearing, and early death. Fifty-three years later, the first large cohort of patients was described by Hagberg et al. [28], which posed similar findings to those published by Krabbe (1916). In Hagberg’s study, clinical evaluations were performed in different regions of Sweden, and patients were assessed by different physicians. The study reviewed the records of 90 patients who were suspected to have the diagnosis of GLD and concluded that 32 of these patients had early-infantile Krabbe disease. In 2009, Duffner et al. conducted a registry-based study, which described the average age at onset, initial symptoms, survival, and clinical features of Krabbe disease from responses of 334 parent questionnaires. A follow up study, focusing specifically on patients with onset between 0 and 6 months, was published in 2011 and expanded its dataset to include sporadic medical records and parent questionnaires of 67 patients who were evaluated at multiple sites and by different physicians. Continuing to expand their use of registry data, Duffner and colleagues published a third study in 2012, which described 45 patients with onset after 6 months of age, including 12 patients with onset between 7 and 12 months, 27 patients with onset between 13 months-10 years, and 6 patients with onset after > 10 years [29, 30]. Our group recently published a study showing prospective data on patients with late infantile Krabbe disease, on 35 patients with Krabbe disease, which together describes in detail 123 patients [31].

In light of the lack of prospective studies, the purpose of this research was to longitudinally describe the neurological progression including signs, symptoms, and neurodevelopmental function in children diagnosed with Krabbe disease and onset between the ages of 0–5 months. Patients were evaluated at a single site, examined by two neurodevelopmental pediatricians, and followed throughout the course of their disease. Multiple standardized tests in all areas of development were performed using a prospectively designed protocol, allowing for internal consistency across patients and evaluations. Growth parameters were measured at every visit, and brain magnetic resonance imaging (MRI) and other neurophysiological analyses were carried out at baseline and longitudinally, when appropriate. Altogether, this is the largest prospective study investigating the natural history of Krabbe patients with onset between 0 and 5 months.

## Methods

### Inclusion/exclusion criteria

From September 1999 to September 2018, 99 Krabbe patients with onset between 0 and 5 months were referred to the Program for the Study of Neurodevelopment in Rare Disorders (NDRD) at Children’s Hospital of Pittsburgh and at University of North Carolina for evaluation, disease management, and/or enrollment in a natural history study. Diagnosis was made by measuring GALC activity in white blood cells or fibroblasts, performed at the Lysosomal Diseases Testing Laboratory at Jefferson Medical College, and were confirmed by GALC gene analysis. However, since mutation analysis was not used consistently for diagnosis until approximately 2009, most patients diagnosed prior to 2009 lack genetic data. Children who underwent HSCT were censored on the day they began the transplantation protocol.

Eighty-eight of the 99 patients screened met the inclusion criteria for the prospective protocol. Eleven patients were excluded either because parents failed to give informed consent (*n* = 1), the patient was transplanted prior to their first visit to the NDRD (*n* = 7), or a full evaluation could not be completed due to extenuating circumstances (*n* = 3). The study was approved by the institutional review boards of the University of North Carolina (IRB-08-0237) and the University of Pittsburgh (IRB-PRO11050036).

### Data collection and analysis

Parents completed a detailed medical history questionnaire, and records received from outside healthcare providers were reviewed. Each patient was evaluated by a multidisciplinary team that included a neurodevelopmental pediatrician, psychologist, speech pathologist, audiologist, ophthalmologist, and occupational and physical therapist. Patients were evaluated longitudinally to monitor the progression of neurologic disease and provide recommendations for disease management. Standardized tests were administered to differentiate ability level between developmental domains. The transition to age-appropriate assessment tools was done according to the patient’s estimated developmental age, and outcomes were compared to the norms of typically developing children [32–37]. Disease-related variables were acquired from parent questionnaires and patient medical records. Survival data was obtained by parent report, internet searches and/or querying the United States social security death index database. The Kaplan-Meyer method was used to estimate the median and overall survival time until November 1, 2018.

In order to track the progression of disease, patients were divided into age groups based on the age at which their evaluations occurred. While patients who were evaluated once only contribute data to a single age group, others, who have been followed longitudinally, have multiple evaluations at different ages; therefore, data from such patients may be included at multiple time points. See Table 1 for a detailed breakdown of overall symptom incidence within each age group.

**Table 1:** Symptom incidence in each age group and cohort as a whole

	0-3 months (*n* = 16)		4-6 months (*n* = 28)		7-9 months (*n* = 36)		10-12 months (*n* = 21)		13-17 months (*n* = 19)		18-23 months (*n* = 11)		>24 months (*n* = 16)	
Symptom	N	Mean	N	Mean	N	Mean	N	Mean	N	Mean	N	Mean	N	Mean
Reflux	12	17%	26	**62%**	25	**84%**	18	**61%**	8	**50%**	5	40%	11	45%
Feeding Difficulties or swallowing difficulties	14	7%	28	**79%**	33	**97%**	21	**90%**	16	**88%**	8	**100%**	12	**100%**
Required Suctioning	12	0%	19	11%	28	**50%**	18	**50%**	16	**81%**	11	**91%**	15	**87%**
Diarrhea	10	0%	20	25%	20	10%	16	6%	12	0%	6	0%	12	17%
Constipation	10	10%	21	38%	29	**59%**	18	**56%**	16	**63%**	10	70%	16	**75%**
Visual Tracking Difficulty	14	7%	28	**57%**	33	**58%**	19	**68%**	19	**89%**	11	**64%**	16	**88%**
Jerky eye movements/eye fluttering	13	0%	17	12%	26	27%	19	32%	14	29%	9	**78%**	13	**54%**
Nystagmus	12	0%	17	12%	24	0%	18	17%	18	22%	10	20%	14	21%
Hip asymmetry	12	0%	15	7%	25	12%	21	24%	19	47%	11	**55%**	15	**87%**
Scoliosis	10	0%	16	13%	28	14%	20	40%	17	**65%**	10	**70%**	16	**94%**
Axial Hypotonia	12	17%	23	**65%**	30	**83%**	21	**100%**	19	**100%**	11	**100%**	15	**100%**
Abnormal DTRs	12	17%	26	**88%**	31	**94%**	21	**95%**	19	**100%**	11	**100%**	15	**100%**
Clasped Thumb/Hand Fisting	12	33%	26	**92%**	32	**94%**	20	**85%**	18	**78%**	10	**60%**	15	40%
Appendicular Spasticity	13	8%	24	**100%**	34	**97%**	20	**90%**	19	**74%**	11	**64%**	16	**75%**
Clinical Seizures	10	0%	18	6%	25	8%	20	15%	16	19%	9	33%	15	33%
Staring Episodes	10	0%	17	41%	27	48%	17	41%	14	**64%**	9	**89%**	12	**92%**
Shallow Breathing or Cheyne-Strokes	12	0%	24	17%	30	37%	21	48%	19	**79%**	11	**82%**	16	**88%**
Apneic Episodes	12	0%	17	18%	26	23%	19	32%	17	**59%**	10	**60%**	16	**63%**
Poor Peripheral Perfusion	11	0%	18	0%	24	4%	18	17%	18	44%	10	**60%**	14	**71%**
Temperature Instability	14	0%	28	0%	34	6%	21	14%	19	32%	11	**55%**	16	**69%**
Abnormal Pupillary Response	11	0%	23	22%	30	43%	19	**58%**	19	**89%**	11	**100%**	14	**100%**

Displays the incidence of each symptom for patients evaluated between 0 and 3 months, 4–6 months, 7–9 months, 10–12 months, 13–17 months, 18–23 months, and ≥ 24 months. Symptoms with incidence > 50% are bolded. N is the number of patients which exhibited the symptoms at that time point. n is the number of patients that were evaluated for that age range

## Results

### Patient characteristics

We evaluated 88 children with early onset Krabbe disease (38 boys, 50 girls). Twenty-nine patients were followed longitudinally (median number of 3 visits). The remaining 59 children were evaluated only once because they either underwent HSCT after their baseline visit (*n* = 26) or were unable to return for reasons related to travel or financial difficulties. Thirteen patients were identified and diagnosed because of a family history of the disease. For the remaining 75 symptomatic patients, the median age at diagnosis was 6 months (range: 3–15 months). The median age at which parents first noticed symptoms was 4 months (range: 0–6 months). The median delay between symptom onset and diagnosis of Krabbe disease was 3 months (range: 0.5–13 months). A total of 146 evaluations were conducted. To examine the progression of symptoms, the evaluations were grouped by age into 0–3 (*n* = 16), 4–6 (*n* = 28), 7–9 (*n* = 36), 10–12 (*n* = 21), 13–17 (*N* = 19), 18–23 (*n* = 11), > 24 months (n = 16) (Table 1).

### Prenatal history, diagnosis, and neonatal problems

Of the 88 patients 30 (34%) developed neonatal problems (jaundice requiring phototherapy, *n* = 14; breathing difficulties, *n* = 4; hypoglycemia, n = 3; feeding difficulties, *n* = 8; Erb’s palsy, n = 1; irregular heartbeat, *n* = 6; or clavicle fracture due to difficult delivery, n = 2). Thirty-two (36%) infants were delivered by cesarean section (frequency in normal US populatio*n* = 32%).

### Initial symptoms

**Fig. 1:**
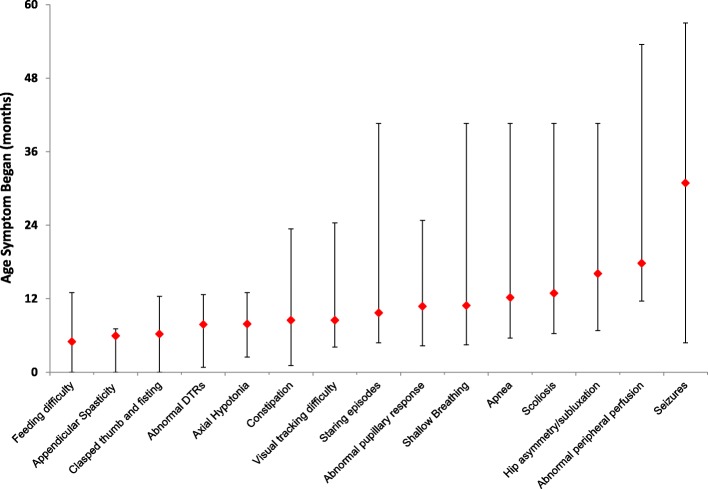
Ages at which common symptoms appear in children with Krabbe disease. The red diamond represents the median age at which the symptom began. The lines show the minimum and maximum ages that the symptom began

Thirteen of the patients were asymptomatic at the time of first evaluation. Of the remaining 75 symptomatic patients, irritability was the most common initial symptom (*n* = 41; 54%), followed by feeding difficulties (*n* = 27; 36%), spasticity (*n* = 25; 33%), reflux (*n* = 17; 23%), developmental delay (*n* = 25; 33%), poor weight gain (*n* = 11; 15%) abnormal movements (*n* = 9; 12%), and decreased tone (*n* = 7; 9%). Other initial symptoms included sleeping difficulties (*n* = 3; 4%), exaggerated startle (*n* = 4; 5%), constipation (*n* = 2; 3%), seizures (*n* = 1; 1%), spasms (*n* = 1), and lethargy (*n* = 1; 1%). Given the prevalence of irritability as the most common initial symptom, we reviewed the onset of this symptom and found that the median age of onset for irritability was 4 months (range: 0–8 months), with 62 (82%) symptomatic patients presenting with irritability by 8 months of age (Fig. 1).

### Growth and survival

**Fig. 2:**
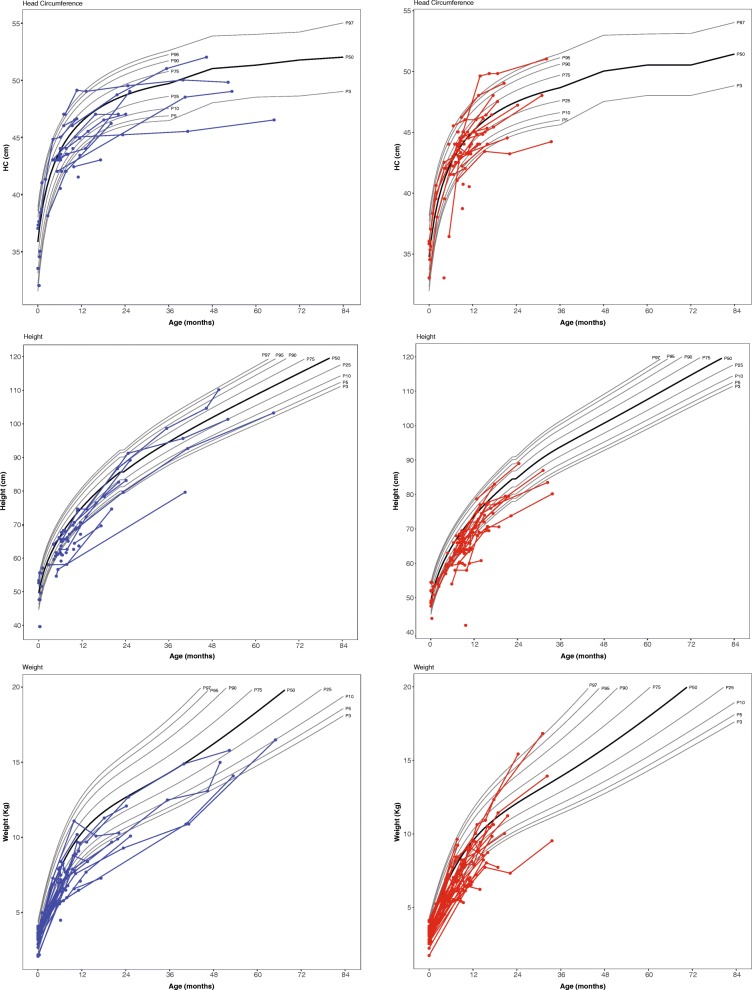
Height, weight, and head circumference of boys (blue) and girls (red) with Krabbe disease. Each circle depicts an individual measurement; lines connecting circles show multiple measurements for an individual child. The gray lines represent standard growth curves (gray lines = 3rd, 5th, 10th, 25th, 50th, 75th, 90th, 95th, and 97th percentiles)

**Fig. 3:**
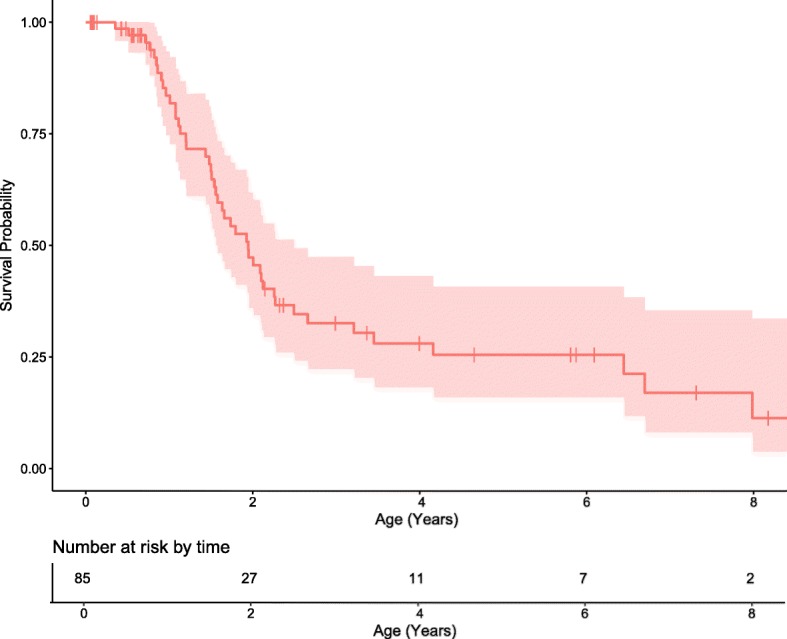
Kaplan–Meier curve of overall survival with a median survival of 2 years. The red shaded area represents the 95% confidence interval. The median survival was 2 years. The x-axis shows age in years and the table below the axis shows the number of patients at risk for an event. The y-axis indicates the cumulative survival to specific age. Each drop in the curve represents a death. The vertical lines along the curve indicate that a child was censored while still living (either because they received UCBT or that they were still living at the cutoff date of 11/01/2018). The cumulative probability is calculated as the product of the interval probabilities up to the age of the event. The denominator for the calculation of the interval probability is the number of children at risk at the time of the event. Children who are censored while living do not contribute to the denominator for the next event

Head circumference was within the normal range for most patients, although 12.5% (*n* = 11) of patients were microcephalic at least one-time point. Height and weight were below the normal range, 28% (*n* = 25) and 31% (*n* = 28), respectively (Fig. 2). Of the 88 patients, 57 are deceased. Results of Kaplan–Meier analysis estimated a median survival time of 2 years. The earliest death occurred at 6 months. Seventy percent of patients were deceased by 2.5 years of age. Twenty percent of patients survived until at least 6 years of age (Fig. 3).

### Feeding and gastrointestinal problems

Gastroesophageal reflux, one of the more common features of this disease, was present in 17% of patients evaluated between 0 and 3 months, 62% of patients evaluated between 4 and 6 months old and 84% evaluated between 7 to 9 months. After 10 months of age, the number of patients with reflux began to decline, likely due to earlier treatment with Nissen fundoplication surgery and antireflux medications.

One baby (7%) evaluated from 0 to 3 months of age had feeding problems and difficulty swallowing. However, the proportion of patients with feeding difficulties increased to 79% in patients evaluated after 4 months of age and to 97% of patients after 7 months of age. To meet nutritional requirements and avoid complications (e.g., aspiration pneumonia), gastrostomy tube placement with a Nissen fundoplication was recommended. Seventy percent of children evaluated from 10 to 12 months, and 100% of children who were evaluated after 12 months of age had surgery for gastrostomy tube placement. Forty-two percent of the patients examined between 10 and 12 months, and 100% of patients evaluated after 24 months had undergone Nissen fundoplication. Gastrointestinal problems were evident in a large number of patients and included constipation and diarrhea. Diarrhea was most common in patients evaluated between 4 and 6 months of age (25%). Constipation was noted in only one patient (10%) aged 0–3 months; however, the incidence of constipation became more common as the disease progressed and was observed in 81% of patients evaluated after 24 months. The need for suctioning due to the inability to manage secretions was apparent in 50% of patients evaluated between 7 and 9 months of age; however, by 18 to 23 months of age, the need for suctioning increased to 91%.

### Vision and hearing

Data showed that visual tracking difficulties increased with disease progression, with 93% of patients evaluated after 24 months exhibiting severe or complete inability to visually track. Abnormal eye movements, such as eye fluttering or jerking, were most common in patients evaluated between 18 and 23 months of age (78%). Parental concern for vision loss was reported in 9% of patients (median: 16 months; range 4.8–54 months). However, visual evoked potentials (VEP), which were considered abnormal if the P100 wave was absent, confirmed visual pathway neuropathy in the majority of the sample. Specifically, 60% of patients tested between 0 and 3 months had abnormal VEP results, including a 5 days old baby. The percentage of abnormal VEP increased to 100% for patients older than 18 months of age. As for hearing, 2% of parents reported hearing loss. However, auditory neuropathy, identified through auditory brainstem responses (ABR), revealed that 70% of patients evaluated between 0 to 3 months had abnormalities in waves, I, III, or V, with this proportion quickly increasing to 100% after 10 months of age.

### Orthopedic complications

Fifty-five percent of the children evaluated between 18 and 23 months and 80% of patients evaluated after 24 months presented with hip asymmetry. Scoliosis increased from 40% in patients evaluated between 10 and 12 months of age to 94% in patients > 24 months.

### Neurologic symptoms and signs

More than 77% of children evaluated after 7 months lacked head control and were unable to sit independently. Mobility became significantly more limited as the disease progressed, with 80% of patients developing quadriparesis between 10 and 12 months of age, and 100% by 24 months. Axial hypotonia was observed in 65% of patients aged 4 to 6 months, 80% of patients evaluated between 7 to 9 months, and 100% in patients evaluated after 10 months of age.

Eighty-eight percent of patients evaluated after 4 months of age had abnormal deep tendon reflexes, which affected 100% of patients by 13 months of age. Clasped thumb and fisting of hands, the most common symptom in patients evaluated between birth to 3 months, began to decrease after 9 months of age. Appendicular spasticity was present in 100% of patients evaluated between 4 and 6 months; however, appendicular spasticity began to decrease starting at 7 months of age and was only present in 70% of patients evaluated after 24 months of age.

Bulging fontanelle was reported in 3% of patients, one of which required a ventriculoperitoneal shunt. Staring episodes were absent in patients younger than 3 months but were present in 83% of patients over the age of 24 months. Clinical seizures were infrequent in children younger than 10 months of age. After 24 months, 40% presented with clinical seizures.

### Autonomic nervous system involvement

Abnormal sweating was reported in 3% of patients (onset: 5, 12, and 18 months). Apneic episodes and changes in heart rate were first reported in patients older than 5 months, and increased in frequency with disease progression. Shallow breathing was present in 17% of patients evaluated between 4 to 6 months, 37% of patients evaluated between 7 to 9 months and increased to 79% in patients evaluated between 13 and 17 months and 88% in patients > 24 months. Of the four (4%) patients who had Cheyne–Stokes respiration, one was 6 months old and three were > 12 months. Poor peripheral perfusion was noted in patients evaluated after 8 months of age, with this sign present in 70% of patients evaluated after 24 months.. Mottling of skin was observed in 28% of patients evaluated after 8 months. Temperature instability was reported in children older than 8 months and became more frequent over time (69% in patients older than 24 months). Results of physical examinations showed delayed or absent pupillary response in patients beginning at 4 months, increasing to 89% incidence in patients evaluated between 13 and 17 months, and reaching 100% in patients evaluated after 18 months of age. Although pinpoint pupils were the most common finding, some patients also presented with dilated pupils or anisocoria.

### Neuroradiologic and neurophysiologic testing

Only two patients (14%) evaluated between 0 and 3 months had normal brain MRI scan readings; 100% of scans after 4 months of age were abnormal. Of the 50 EEGs available for analysis, 52% were normal, with generalized slowing evident in 40% of patients. Results of NCV studies showed that 78% of patients tested between 0 and 3 months and 95% between 4 and 6 months had abnormal motor nerve conduction studies. 100% of patients had abnormal motor nerve conduction by 6 months of age. Abnormal sensory nerve conduction was observed in 93% of all patients, reaching 100% by 7 months of age.

### Cerebrospinal fluid and IgG index

**Fig. 4:**
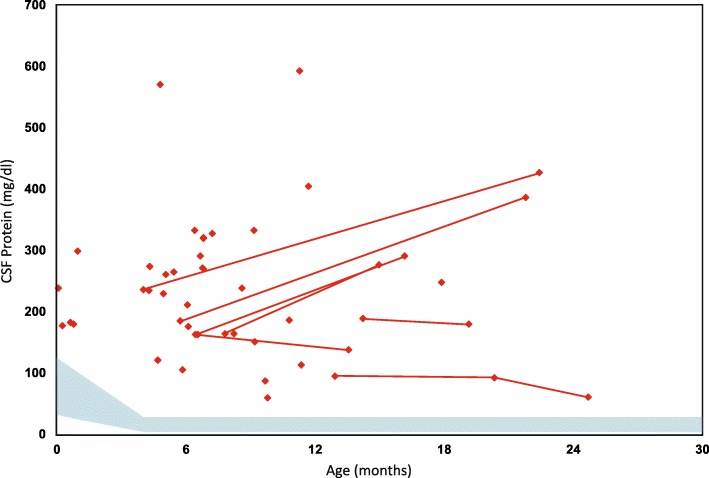
CSF protein levels. CSF protein levels were plotted against patient age. The red diamonds represent the CSF protein level at a single point in time. Levels from the same individual, but at different points in time, are connected by red lines. The blue shaded area represents 95% variability of CSF protein level of the normal population by age

Hyperproteinorrachia was detected in 100% patients who were tested [38, 39]. There were 70 cerebrospinal fluid (CSF) protein values with a median of 208 mg/dl and ranged from 43 to 594 (Fig. 4). CSF protein levels were especially high in two patients (> 550 mg/dl); however, higher protein levels did not correlate with clinical manifestations, disease severity or genotype. No correlation between gross motor scores or onset of symptoms with protein levels in CSF was found (*p* > 0.39).. The CSF IgG index was normal (< 0.85) in all patients who were tested. The albumin index, which predicts blood–brain barrier damage, was elevated in 11 of 30 available results but did not correlate with disease severity (CSF IgG index range: 0.006–0.092; median 0.040).

### Genetic analysis and enzyme activity

GALC gene analysis was available for 39 patients. Genetic variants, age of onset, and initial symptoms for these patients are displayed in Table 2.

**Table 2:** Genotype/Phenotype correlation for patients (*n* = 39) with available data

Patient	Allele 1		Allele 2		Age at Onset (Months)	Earliest Clinical Symptoms
	DNA	Protein	DNA	Protein		
1	c.1586C>T	p.Thr529Met^#2^	c.1586C>T	p.Thr529Met^#2^	4	Feeding difficulty; Irritability
2	g.30Kbdel		c.1158del10	p.Met387Phefs*?	0	Irritability
3	g.30Kbdel		c.622-1G>T	Missplicing	NA	Asymptomatic
4	c.242_243dupGA	p.Ile82Argfs*?	c.1700A>C	p.Tyr567Ser^#1^	4	Developmental delay; Spasticity
5	c.379 C>T	p.Arg127*	c.864G>A	p. Trp288*	4	Spasticity; Feeding difficulty; Irritability
6	g.30Kbdel		g.30Kbdel		3	Feeding difficulty
7	g.30Kbdel		c.430delA	p.Ile144Leufs*?	3	Spasticity; Feeding difficulty
8	c.628A>T	p.Arg210*	c.1895T>C	p.Leu632Pro^#1^	3.5	Spasticity; Feeding difficulty; Irritability
9	c.884A>T	p.Asn295Ile^#1^	c.884A>T	p.Asn295Ile^#1^	1	Feeding difficulty
10	g.30Kbdel		c.544G>A	p.Glu198Lys	4	Irritability
11	g.30Kbdel		c.658C>T	p.Arg220*^#1^	1.5	Feeding difficulty; Irritability; Reflux
12	c.749T>C	p.Ile250Thr	c.1586C>T	p.Thr529Met	4	Feeding difficulty; Reflux
13	c.1884delA	p.Lys628Asnfs*7^#2^	g.5.78 Mb del		3	Feeding difficulty; Irritability; Reflux
14	c.155delG	p.Gly52Alafs*?	c.155delG	p.Gly52Alafs*?	3	Feeding difficulty; Irritability; Reflux
15	g.30Kbdel		c.620delA	p.Lys191fs*?	2.5	Feeding difficulty; Developmental delay
16	c.430delA	p.Ile144Leufs*?	c.628A>T	p.Arg210*	5	Spasticity; Irritability; Constipation
17	g.30Kbdel		c.1657G>A	p.Gly553Arg	4	Spasticity; Irritability; Developmental delay
18	g.30Kbdel		g.30Kbdel		2	Irritability; Developmental delay; Poor weight gain; Spasms
19	c.583-6T>A^#1^	Missplicing	g.7.4Kbdel^#1^		5	Feeding difficulty; Developmental delay; Hypotonia
20	c.2056T>C	p.*686Glnext*42^#1^	c.430DelA	p.Ile144Leufs*?^#1^	3	Developmental delay
21	c.316G>A	p.Gly106Arg	c.521delA	p.Tyr158Leufs*3	4	Irritability
22	c.967G>T	p.Gly323Trp	c.1472delA	p.Lys491Argfs*62	4	Irritability; Reflux
23	c.489G>A	p.Trp163*^#1^	c.489G>T	p.Trp163Cys^#1^	5	Spasticity; Irritability; Developmental Delay
24	c.387C>G	p.Tyr129*	c.1814dupA	p.Tyr605fs*1	5	Spasticity; Developmental delay
25	c.533G>A	p.Trp178*^#1^	c.1468T>A	p.Tyr490Asn^#1^	NA	Asymptomatic
26	g.30Kbdel		c.674C>A	p.Ala225Glu	NA	Asymptomatic
27	g.30Kbdel		g.30Kbdel		3	Feeding difficulty; Hypotonia
28	g.30Kbdel		c.1004A>G	p.Tyr335Cys^#1^	3	Feeding difficulty; Spasticity
29	g.30Kbdel		c.1004A>G	p.Tyr335Cys^#1^	4	Spasticity; Irritability; Developmental Delay; Hypotonia
30	g.30Kbdel		c.490C>A	p.Pro164Thr	5	Spasticity; Developmental delay
31	g.30Kbdel		g.30Kbdel		4	Spasticity; Developmental delay
32	c.1700A>C	p.Tyr567Ser^#1^	c.1158del10	p.Met387Phefs*?	2	Reflux
33	g.30Kbdel		c.1543G>A	p.Glu515Lys^#1^	5	Irritability; Reflux
34	c.764C>A	p.Pro255His^#1^	c.1591C>T	p.Arg531Cys^#1^	1	Spasticity
35	g.30Kbdel		c.1004A>G	p.Tyr335Cys^#1^	5	Feeding difficulty; Spasticity; Developmental delay
36	g.30Kbdel		g.30Kbdel		5	Spasticity; Irritability
37	g.30Kbdel		g.30Kbdel		5	Feeding difficulty; Developmental delay
38	g.30Kbdel		c.1766dupA	p.Tyr605fs*1^#1^	4	Developmental delay; Hypotonia
39	c.869G>A	p.Arg290His	c.1480T>C	p.Phe494Leu	4	Development delay: irritability

Patients 3, 25 and 26 were transplanted before symptoms. Variants are reported using Human Genome Variation Society (HGVS) nomenclature. Reference sequences NP_000144.2 (Protein) and NM_000153.3 (cDNA nucleotide). The most common infantile-onset allele, g.30Kbdel (c.1161 + 6555_*9573del), was always detected in ‘cis’ with the pseudodeficiency variant p.Arg184Cys (c.550C > T). ^#^variants detected in ‘cis’ with the pseudodeficieny alleles p.Ile562Thr (c.1685 T > C)^#1^ or p.Asp248Asn (c.742G > A)^#2^

DNA was extracted from 3-mm dried blood spots (DBS) using the CASM method [40]. The molecular test was performed using the polymerase chain reaction (PCR) followed by fluorescence-based (Sanger) sequence analysis of the 17 coding exons, intron/exon boundaries and the promoter region of the GALC gene. Testing for g.30kbdel, the most common infantile-onset GALC pathogenic variant, and g.7.4del, was performed using gap-PCR and gel analysis.

All GALC enzyme activity levels were low, with a median value of 0.05 nmol/h/mg protein (range: 0–0.3 nmol/h/mg protein).

### Neurodevelopmental function

**Fig. 5:**
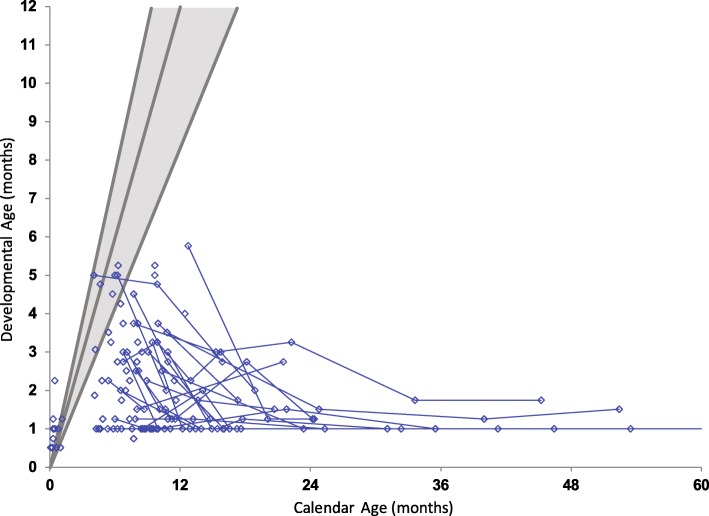
Cognitive development in children with Krabbe disease. Age-equivalent scores (i.e., developmental age) are graphed against actual age for cognitive development. Evaluations performed on the same individuals, longitudinally, are connected by blue lines. The gray lines from left to right represent the approximate 97.5th, 50th and 2.5th percentiles of development for normal age-matched controls

**Fig. 6:**
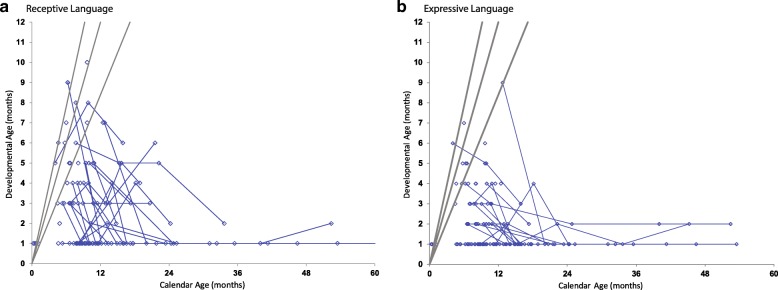
Language development in children with Krabbe disease. Age-equivalent scores (i.e., developmental age) are graphed against actual age for (**a**) receptive and (**b**) expressive language. Evaluations performed on the same individuals, longitudinally, are connected by blue lines. The gray lines from left to right represent the approximate 97.5th, 50th and 2.5th percentiles of development for normal age-matched controls. Because of limitations in the Mullen Scales of Early Learning, the lowest AE a patient could score is 1 month

**Fig. 7:**
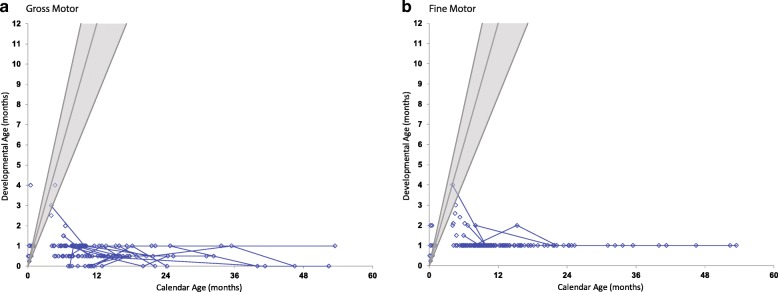
Motor development in children with Krabbe disease. Age-equivalent scores (i.e., developmental age) are graphed against actual age for (**a**) gross and (**b**) fine motor. Evaluations performed on the same individuals, longitudinally, are connected by blue lines. The gray lines from left to right represent the approximate 97.5th, 50th and 2.5th percentiles of development for normal age-matched controls

Cognitive function, expressive and receptive language, and motor development were evaluated for each patient. All children who were evaluated longitudinally showed rapid developmental regression in multiple domains. Patients who achieved higher scores on follow up testing than baseline testing in one or more domains were either extremely irritable at baseline testing, which subsided after medication management by the time of their follow up evaluation or were still asymptomatic at the time of their baseline evaluation (Figs. 5, 6, and 7).

Cognitive function was assessed using standardized protocols that tested the ability to listen, solve visual problems, and perform simple tasks. None of the patients scored higher than 6 months age equivalence at any time point, and by 6 months of age, all patients fell below the normal range (Fig. 5).

Receptive language was the strongest area of development and had the largest number of patients scoring within the normal range; nevertheless, all patients were below normal in receptive language by 10 months of age. Most patients scored comparatively worse in expressive language (Fig. 6).

Gross motor function was the weakest area of development. All asymptomatic patients had normal gross motor function, whereas all symptomatic patients had below normal function, with most having skills of a 1 month or less age equivalency after 5 months (Fig. 7). In regard to fine motor skills, most symptomatic children scored at a 1-month old level after 8 months, while asymptomatic children scored in the average or above average range.

## Discussion

In this study, we prospectively evaluated a large cohort of Krabbe patients with onset between 0 and 5 months. Unlike earlier studies, such as Duffer et al. (2011) and Hagberg et al. (1969), the current study implemented a prospective longitudinal design, in which patients were evaluated at a single site by two neurodevelopmental pediatricians working with a multidisciplinary team using standardized tests. To our knowledge, this is the largest single site prospective study characterizing the natural history of early onset Krabbe disease. Results of the study reveal a disease course, in which the majority of patients are minimally symptomatic or asymptomatic from 0 to 3 months of age. However, results of MRI, and NCVs revealed evidence of disease much earlier [41, 42]. Nevertheless, once clinical symptoms emerged, disease progression was rapid and severe.

Given the rapid progression of disease following onset, it is imperative that physicians can identify early clinical markers of disease. Overall, we found irritability, clasped thumb and hand fisting to be the most common indicators of disease in patients aged 0–3 months of age. Although reflux was prevalent in these young patients, it did not exceed the incidence of reflux in the normal age-matched population until after 7 months of age [43, 44]. Interestingly 100% of NCV were abnormal by 6 months of age. For patients aged 4–6 months and patients aged 7–9 months, the most prevalent markers of disease were irritability, clasped thumbs and hand fisting, appendicular spasticity, feeding and swallowing difficulty, and abnormal DTR’s. In patients aged 10–12 months, axial hypotonia was evidenced. For patients 13–17 months of age, markers such as staring episodes, shallow breathing, visual tracking difficulties, and abnormal pupillary response began to evolve. However, it should be noted that a large decrease in the incidence of appendicular spasticity was observed in patients > 12 months, likely attributable to progression in peripheral nerve disease resulting in severe muscle weakness. Patients over the age of 18 months, developed in addition of eye fluttering, severe constipation, orthopedic complication (i.e. scoliosis and hip subluxation), and dysautonomia (i.e. temperature instability, non-pitting edema, and poor peripheral perfusion), all of which become more prevalent in children more than 2 years of age.

Despite the severe and rapid disease progression throughout the first 2 years of life, our group has previously demonstrated that HSCT treatment in babies within the first 2 months of life (when they are mostly asymptomatic) is associated with essentially normal receptive language skills, continuous gains in cognitive skills, extended survival, and improvement or stabilization in brain atrophy, as shown by brain MRI scans [16, 20]. Unfortunately, babies become symptomatic within 4 months after birth but are not diagnosed in a timely manner. The majority are ineligible for HSCT because their disease may already be too advanced [16, 18, 45]. Thus, earlier diagnosis will allow more patients to benefit from treatments that substantially reduce disease progression and improve their quality of life. Although extreme irritability appears early and is the most common initial symptom of disease, irritability due to colic is also typical in the normal pediatric population beginning around 4 months of life, making it common for physicians and caretakers to initially disregard the concern. However, unlike colic, irritability in Krabbe disease is much more severe and constant throughout the day [46]. Therefore, we strongly recommend that severely irritable babies undergo a feeding evaluation, and a neurological examination. For babies who present with abnormal muscle tone, slow feeding, swallowing difficulties, and/or reflux, we recommend a brain MRI. Abnormal findings should prompt subsequent enzyme activity and genetic analysis [31].

Although recognizing the early signs of infantile Krabbe disease frequently delays diagnosis and prevents effective HSCT, NBS will allow for the early diagnosis and treatment. NBS was initiated in New York on August 7, 2006, started in Missouri in August 2012, Kentucky and Ohio in 2016, Illinois and Tennessee since 2017. Legislation to screen for Krabbe disease has since been passed in the states of Louisiana, New Jersey, New Mexico, and Pennsylvania. Nevertheless, natural history data will remain important, as NBS is not available in many states and our understanding of the genetic and biochemical markers of Krabbe disease are still limited. While GALC enzyme activity has been used to diagnose the disease, the correlation between enzyme activity and phenotype remains poor. Increased psychosine levels in dry blood spots (DBS) are currently being used as a second-tier biomarker that can accurately predict early disease onset [25]. However, further work is necessary to understand the relationship between natural disease progression, pathogenic variants in the GALC gene, enzyme activity, and psychosine levels. Establishing correlations between the genetic variations, psychosine levels and phenotype will require a very large database. This is due to more than 200 known pathogenic, likely pathogenic or variants of unknown significance (VUS) and the multiple pseudodeficiency variants that influence enzyme activity (haplotype effect) when in ‘cis’ with a ‘mild’ allele [47, 48]. Thus, until these databases are further developed, NBS legislation is expanded, and NBS methodologies are refined, awareness and knowledge of the presenting symptoms and clinical manifestations of early onset Krabbe disease will remain the primary source of early diagnosis and referral for future clinical trials.

Limitations of our study include restricted accessibility to some outside medical records and difficulty recruiting patients for longitudinal follow up once they became too sick to travel. Because a portion of the information was collected via a parent questionnaire, some data is subject to recall bias. In addition, many children underwent HSCT after their first evaluation and therefore could not be followed longitudinally. Because certain symptoms were treated following baseline visits (i.e. reflux, feeding and swallowing difficulties, excretions, diarrhea, and constipation) the incidence of these symptoms in our populations may be slightly altered in respect to the disease’s true natural history. Despite these limitations, the prospective design and standardized protocols for physical and neurodevelopmental evaluations make this study uniquely powerful in providing a comprehensive description of the natural history of patients with early onset Krabbe disease.

## Conclusion

This study characterized the disease progression in Krabbe patients with onset between 0 and 5 months of age. Patients presented with extreme irritability and feeding difficulties. However, evidence of disease was apparent on MRI, NCVs, ABRs and VEPs well before patients became clinically symptomatic, which is especially important in asymptomatic children with low enzyme level and likely pathogenic variants or VUS that are being monitored for disease onset after screening positive through NBS programs. Because of the haplotype effect, reporting the pseudodeficiency allele is important. As the disease progressed, patients developed appendicular spasticity and abnormal DTR’s, followed by axial hypotonia, visual tracking difficulties, staring episodes, quadriparesis, scoliosis, and dysautonomia. An improved understanding regarding the clinical course of this rapidly progressing disease is essential to facilitating early diagnosis, assisting in the management and treatment of afflicted patients, and designing successful clinical trials.
